# Discovery of a Marine *Beauveria bassiana* Polysaccharide with Antiviral Activity Against Tobacco Mosaic Virus

**DOI:** 10.3390/md24010039

**Published:** 2026-01-13

**Authors:** Xu Qiu, Lihang Jiao, Jingjing Xue, Guangxin Xu, Xixiang Tang

**Affiliations:** 1 Key Laboratory of Marine Genetic Resources, Third Institute of Oceanography, Ministry of Natural Resources, Xiamen 361005, China; qiuxu@tio.org.cn (X.Q.); 17807029891@163.com (L.J.); jjxue0126@163.com (J.X.); xuguangxin@tio.org.cn (G.X.); 2 College of Ocean Food and Biological Engineering, Jimei University, Xiamen 361021, China

**Keywords:** *Beauveria bassiana*, extracellular polysaccharide, Tobacco mosaic virus, *Nicotiana benthamiana*, antioxidant defense, marine fungi

## Abstract

Tobacco mosaic virus (TMV) threatens crop yield and quality, while chemical antivirals offer limited efficacy and potential environmental hazards. Marine fungal polysaccharides are promising eco-friendly alternatives due to their biocompatibility and biodegradability. Here, extracellular polysaccharides (EPSs) from the deep-sea fungus *Beauveria bassiana* T2-2 was isolated, characterized, and produced under optimized conditions (28 °C, 200 rpm, 9 days, pH 8, inoculum 4%) using an L9 (3^4^) orthogonal medium, yielding 3.42 g/L, which is a 48% increase over unoptimized culture. EPSs were glucose-rich, with a molecular weight of 3.56 × 10^4^ Da, containing 90.05% total sugar, 0.28% protein, 1.15% uronic acid, and 1.18% sulfate. In a *Nicotiana benthamiana*–TMV model, EPSs alleviated viral symptoms, maintained chlorophyll content, enhanced antioxidant enzymes (SOD, POD, CAT), reduced malondialdehyde, and upregulated defense genes in SA, ET, ROS, and phenylpropanoid pathways. EPSs, alone or combined with Ribavirin, activated multi-pathway antiviral immunity, highlighting its potential as a sustainable plant-protective agent.

## 1. Introduction

Plant viruses cause severe damage to plant growth and morphology, resulting in symptoms such as stunted growth and fruit deformation, and in serious cases, plant death. These infections greatly reduce crop yield and quality, thus limiting agricultural productivity and economic benefits. Among them, tobacco mosaic virus (TMV) is one of the most prevalent and destructive plant viruses, often referred to as “plant cancer” [1]. It has been estimated that TMV alone causes global agricultural losses of up to USD 100 million annually [2]. In tobacco production, TMV has become the most damaging pathogen, surpassing fungal and bacterial diseases [3].

Current TMV control strategies mainly adopt an integrated approach, emphasizing “prevention and comprehensive management,” which includes cultivation of resistant varieties, improved agricultural practices, and application of antiviral chemicals to induce host resistance or inhibit viral replication [4,5]. Although chemical control offers rapid and convenient effects, there is still no pesticide that effectively suppresses viral infection without harming the host plant [6]. Common antiviral agents such as ribavirin and ningnanmycin exhibit only moderate efficacy against TMV, and chemical treatments also pose potential environmental and health risks. In this context, biological control using natural metabolites from microorganisms or plants has gained increasing attention for its environmental friendliness, safety, and cost-effectiveness [7]. However, developing efficient and eco-friendly antiviral agents remains a major challenge.

Polysaccharides are natural high-molecular-weight polymers composed of ten or more monosaccharide units linked by glycosidic bonds, widely distributed in yeasts, bacteria, algae, grains, legumes, and medicinal fungi [8]. Fungal polysaccharides represent an important class of biopolymers, existing either in the cell wall or secreted extracellularly as defensive or adhesive materials [9]. Their composition and activity vary with fungal species and cultivation conditions [10]. Many natural polysaccharides have shown antiviral potential. For example, the neutral polysaccharide LNT and its sulfated derivative sLNT from *Lentinus edodes* displayed strong inhibitory activity against TMV infection [11]. Fructooligosaccharides from burdock induced the expression of resistance-related genes, including pathogenesis-related (PR) and antioxidant enzyme genes, enhancing TMV resistance in tobacco [12]. The neutral polysaccharide DNPE6(4) isolated from *Dendrobium nobile Lindl.* triggered calcium signaling and defense protein expression, improving protection against pathogens [13]. Compared with chemical agents, polysaccharides are non-toxic, biodegradable, and leave no harmful residues, showing great promise as environmentally friendly antiviral agents. Fungal polysaccharides, in particular, are renewable, biocompatible, and degradable, and are widely used in pharmaceuticals, food, and biomaterials [14].

The marine environment, covering about 70% of the Earth’s surface, includes deep-sea regions (>1000 m) characterized by high pressure, low temperature, limited light, and oxygen deficiency [15]. Despite these extreme conditions, the deep sea harbors diverse microorganisms with unique metabolic systems. Marine fungi can adapt to such environments through symbiotic or independent lifestyles, producing structurally novel and functionally diverse metabolites [16,17]. Compared with terrestrial fungi, marine-derived fungi often possess special biosynthetic pathways and thus represent an important source of new bioactive compounds.

In this study, the extracellular polysaccharides (EPSs) were isolated from the marine-derived entomopathogenic fungus *Beauveria bassiana* T2-2 obtained from the East Pacific sediment. The physicochemical characteristics and antiviral activity of EPSs against TMV were systematically investigated. Furthermore, its potential mechanism in inducing plant resistance was explored at both physiological and molecular levels. This work aims to elucidate the antiviral mechanism of marine fungal polysaccharides and expand their potential applications as eco-friendly agents for sustainable plant protection.

## 2. Results and Discussion

### 2.1. Morphological Characteristics and Molecular Identification of Beauveria bassiana T2-2

**Figure 1:**
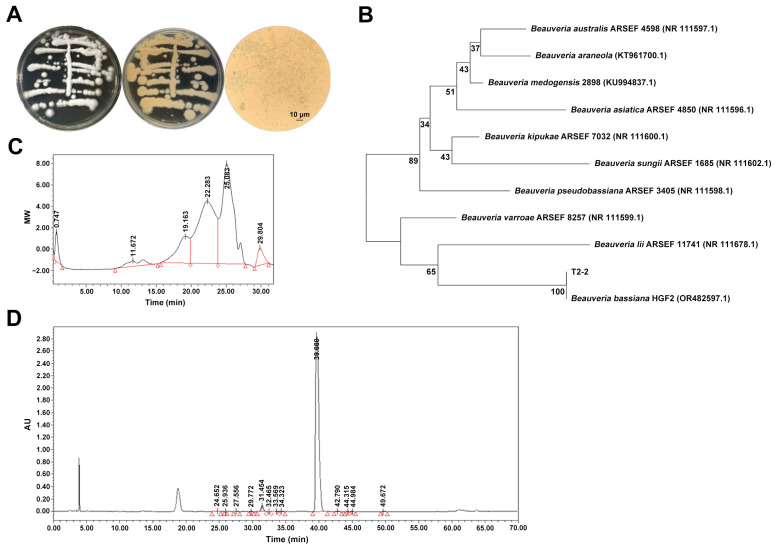
Morphology, phylogenetic identification, and chromatographic characteristics of the EPS-producing strain *B. bassiana* T2-2. (**A**) Colony morphology (obverse and reverse) and hyphal structures of *B. bassiana* T2-2 after 72 h incubation on PDA at 25 °C. (**B**) Neighbor-joining phylogenetic tree based on ITS1-5.8S-ITS4 rDNA sequences showing the taxonomic placement of strain T2-2; bootstrap values > 70% (1000 replicates) are indicated; scale bar = 0.005 substitutions/site. (**C**) HPGPC chromatogram of EPSs detected by refractive index (RI) over a 0–32 min elution window. (**D**) RP-HPLC monosaccharide fingerprint of acid-hydrolyzed EPSs detected at 254 nm.

After incubation on PDA plates at 28 °C for 7 days, strain T2-2 developed white colonies with a slightly raised center and a dense, cottony-to-floccose aerial mycelial layer (Figure 1A). The colony surface was loosely textured, and abundant conidiation formed a conspicuous powdery spore layer. The colony reverse was colorless to pale yellow, consistent with the surface appearance. Microscopic examination revealed hyaline, septate hyphae with characteristic curved growth. Conidia were produced singly or in short chains, and were spherical to sub-ovoid, hyaline, smooth-walled, measuring approximately 2.1–3.0 μm in diameter (Figure 1A).

To clarify its phylogenetic position, the rDNA-ITS region of strain T2-2 was amplified, using the universal fungal primers ITS4/ITS5. BLASTn (https://blast.ncbi.nlm.nih.gov/Blast.cgi) (accessed on 1 November 2025) analysis of the resulting sequence showed 99% identity to *B. bassiana* HGF2 (GenBank accession OR482597.1). In the phylogenetic tree constructed using the neighbor-joining (NJ) method, T2-2 clustered with *B. bassiana* HGF2 in a highly supported monophyletic branch (bootstrap = 100%, Figure 1B). Based on combined morphological and molecular evidence, the isolate was identified as *B. bassiana* T2-2.

### 2.2. Physicochemical Properties and Molecular Weight Distribution of EPSs

Colorimetric assays showed that EPSs consisted predominantly of carbohydrates, with a total sugar content of 90.05 ± 0.87%. Minor amounts of protein (0.28 ± 0.01%), uronic acid (1.15 ± 0.11%), and sulfate (1.18 ± 0.14%) were detected, indicating low impurity levels. These results indicate that the EPSs are glucose-rich glucan-type polysaccharide. Notably, although the sulfate content was low, such substitutions have been reported to influence the bioactivity of marine polysaccharides [18,19].

High-performance gel permeation chromatography (HPGPC) analysis revealed three symmetrical elution peaks (Figure 1C,D), corresponding to components with different degrees of polymerization. Their respective weight-average molecular weights (Mw) were 1.85 × 10^5^ Da (14.30%), 1.71 × 10^4^ Da (49.10%), and 2.00 × 10^3^ Da (36.70%), yielding an overall Mw of 3.56 × 10^4^ Da. This broad molecular weight distribution suggests that EPSs are not a single uniform polymer but instead consist of multiple fractions, a feature common among microbial EPS and often associated with diverse functional properties [20,21].

Monosaccharide analysis revealed a single major peak in the high-performance liquid chromatography (HPLC) chromatogram, whose retention time matched that of glucose (Figure 1D, Figure S1 and Table S1). Glucose accounted for 97.67% of the total molar ratio, while all other monosaccharides together contributed <3.00%, suggesting that EPSs represent glucose-rich polysaccharide fractions rather than fully homogeneous homopolysaccharides. It should be noted that free glucose and other low-molecular-weight sugars derived from the fermentation medium were removed prior to analysis by Sephadex LH-20 gel filtration chromatography [22]. This chromatographic step effectively separates high-molecular-weight polysaccharides from residual monosaccharides based on molecular size. In addition, thin-layer chromatography (TLC) monitoring was performed for all collected fractions, and only polysaccharide-containing fractions were pooled for subsequent analyses (Figure S2). Therefore, the detected glucose originates from acid hydrolysis of the purified polysaccharide fraction rather than from residual fermentation substrate.

### 2.3. Optimization of EPS Fermentation Conditions

#### 2.3.1. Optimization of Fermentation Process Parameters

**Figure 2:**
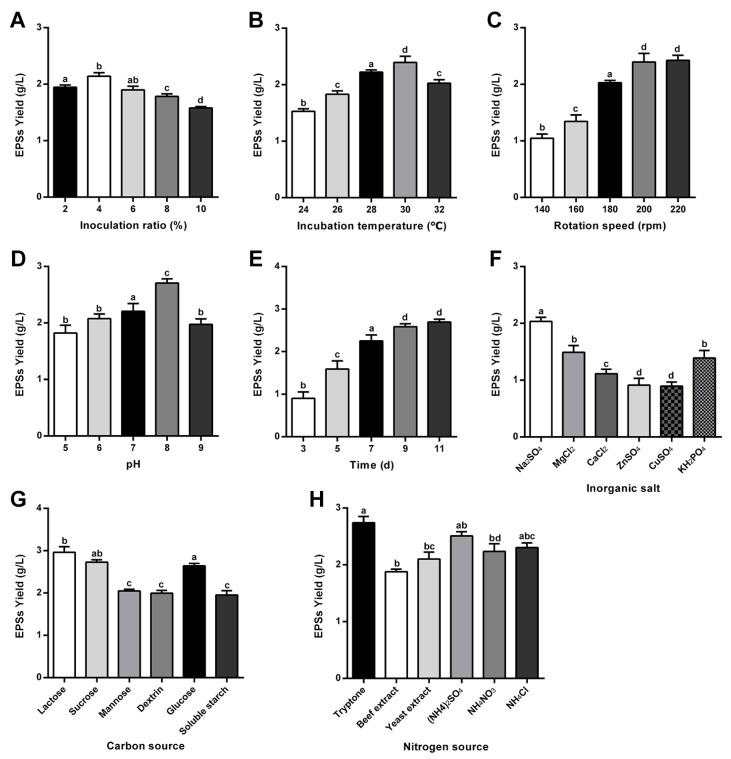
Optimization of fermentation parameters and medium components for EPS production by *B. bassiana* T2-2. (**A**) Inoculum density (%, *v*/*v*). (**B**) Incubation temperature (°C). (**C**) Shaking speed (rpm). (**D**) Initial pH. (**E**) Fermentation duration (d). (**F**) Effects of supplemental inorganic salts. (**G**) Carbon source screening. (**H**) Nitrogen source screening. Data are expressed as mean ± SD (*n* = three biological replicates). Different letters (a, b, c, d) in the figure indicate significance levels. The same letter denotes no significant difference (*p* > 0.05), while different letters indicate a significant difference (*p* < 0.05).

The fermentation parameters significantly influenced EPS biosynthesis by *B. bassiana* T2-2. As shown in Figure 2A, EPS’ yield increased by increasing initial pH below 8, reaching a maximum of 2.70 ± 0.08 g/L at pH 8, followed by a decline at higher pH values. This pattern suggests that pH affects membrane permeability and nutrient uptake, thereby modulating carbon flux into polysaccharide pathways. Similar pH-dependent EPS regulation has been reported in other filamentous fungi, such as the *Penicillium* spp. [23].

Inoculum size also exhibited a clear impact on EPS accumulation (Figure 2B). EPS production peaked at a 4% inoculum (2.14 ± 0.06 g/L). Lower inoculum levels prolonged the lag phase, whereas higher inoculum densities likely increased competition for nutrients and oxygen, leading to reduced polysaccharide synthesis [24]. Thus, 4% was selected as the optimal inoculum size.

Fermentation time influenced both mycelial growth and EPS secretion. EPS accumulated rapidly within the first 7 days, followed by a slower increase between Days 7–9 (Figure 2C). Maximum yield (2.59 ± 0.08 g/L) was observed on Day 9, with no significant difference from Day 11, indicating that EPS production plateaued as cultures entered the stationary phase. Therefore, 9 days was designated as the optimal fermentation duration.

Temperature also played an essential regulatory role. EPS production increased steadily from 24 °C to 30 °C, reaching its highest level at 30 °C (Figure 2D). A further increase to 32 °C caused a decline, likely due to stress responses that redirected metabolic flux away from secondary metabolite synthesis [25].

Agitation speed affected oxygen transfer during fermentation [26]. Although EPS production did not decline within the tested range (Figure 2E), the highest yield was recorded at 220 rpm (2.42 ± 0.09 g/L), with no significant difference compared to 200 rpm (2.39 ± 0.16 g/L). To minimize potential shear stress and energy consumption, 200 rpm was selected as the optimal shaking speed.

#### 2.3.2. Optimization of Medium Components

Among inorganic salts (Figure 2F), Na_2_SO_4_ markedly enhanced EPS production compared with other salts, whereas CuSO_4_ exhibited an inhibitory effect. Sodium sulfate was therefore chosen as the preferred inorganic salt.

Carbon source screening revealed substantial differences among tested substrates (Figure 2G). Lactose supported the highest EPS production, followed by sucrose and glucose, while starch-derived carbohydrates were markedly less efficient. This preference may reflect strain-specific metabolic regulation of sugar uptake and catabolism [27]. Thus, lactose was chosen as the optimal carbon source.

Nitrogen source evaluation (Figure 2H) demonstrated that peptone resulted in the greatest EPS yield among organic nitrogen sources, whereas beef extract supported the lowest production. Among inorganic nitrogen sources, ammonium sulfate performed best. Given their complementary metabolic roles, both peptone and (NH_4_)_2_SO_4_ were selected as optimal nitrogen components.

#### 2.3.3. Orthogonal Design for Integrated Optimization

An L9 (3^4^) orthogonal design was employed to determine optimal medium composition (Table 1). Range analysis indicated that lactose had the greatest effect on EPS production, followed by peptone, (NH_4_)_2_SO_4_, and Na_2_SO_4_. Under these conditions, EPS yield increased to 3.42 ± 0.11 g/L, representing a 48.1% improvement compared with the initial medium (2.31 ± 0.07 g/L).

#### 2.3.4. Overall Optimal Fermentation Conditions

Integrating the results of single-factor and orthogonal analyses, the optimal conditions for EPS production by *B. bassiana* T2-2 were determined as follows: 30 °C, 200 rpm, initial pH 8, 4% inoculum, 9-day fermentation, and medium containing lactose 20 g/L, peptone 3 g/L, (NH_4_)_2_SO_4_ 3 g/L, and Na_2_SO_4_ 0.5 g/L.

### 2.4. Protective Effects of EPSs Against TMV Infection

**Figure 3:**
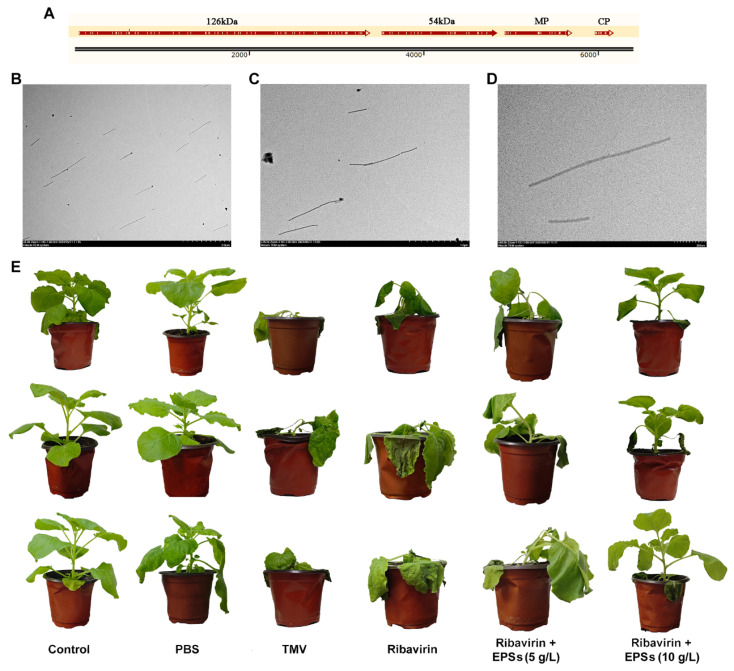
TMV sequence verification, virion ultrastructure, and EPS-mediated protection in TMV-infected *N. benthamiana*. (**A**) Alignment of amplified TMV coat–protein sequences. (**B**–**D**) Transmission electron micrographs of purified TMV particles at 6000× (**B**), 15,000× (**C**), and 40,000× (**D**) magnification, respectively. (**E**) Phenotypic responses of TMV-infected *N. benthamiana* under different treatment conditions.

TMV presence in the extracted virus suspension was confirmed by sequence alignment of four amplified fragments (Figure 3A) with the reference TMV genome (GenBank: AF273221.1) and by TEM visualization of characteristic rod-shaped virions (Figure 3B–D), ensuring the reliability of subsequent plant inoculation.

On Day 7, TMV-inoculated *N. benthamiana* plants exhibited severe wilting, extensive leaf necrosis, and stem lodging, whereas control plants remained healthy (Figure 3E). PBS-treated leaves displayed only minor local necrosis likely caused by mechanical friction. These symptoms collectively confirmed successful TMV infection.

EPS treatment markedly altered disease progression in a dose-dependent manner (Figure 3E). Ribavirin alone did not alleviate disease severity, potentially due to insufficient penetration into actively infected tissues. The co-application of 5 g/L EPSs resulted in mild improvement, while 10 g/L EPSs produced substantial protection, with only the inoculated leaf showing slight wilting and all other leaves remaining free of necrosis. These observations are consistent with previous findings in antiviral filtrates from *Trichosporon*, where filtrate-treated plants showed improved condition compared with TMV-only groups [28].

Mechanistically, EPSs may potentiate plant immunity through activation of the hypersensitive response (HR), which is a localized programmed cell death process that constitutes an early antiviral defense. HR can subsequently trigger systemic acquired resistance (SAR), which is a long-distance signaling mechanism that enhances whole-plant immunity [29,30,31]. The pronounced protective phenotype in the 10 g/L EPS group suggests that EPSs may enhance HR-SAR signaling, thereby mitigating TMV-induced damage.

Collectively, these results demonstrate that EPSs significantly enhances resistance to TMV and synergizes with Ribavirin to reduce symptom severity and improve plant vitality.

### 2.5. EPSs Enhance Antioxidant Defense, Reduce Lipid Peroxidation, and Maintain Chlorophyll in TMV-Infected N. benthamiana

Reactive oxygen species (ROS) regulation is a key component of plant antiviral defense, and antioxidant enzymes such as superoxide dismutase (SOD), peroxidase (POD), and catalase (CAT) cooperate to mitigate oxidative damage and enhance resistance [32]. SOD catalyzes the dismutation of O_2_^−^ to H_2_O_2_, while POD and CAT further convert H_2_O_2_ to water, with POD additionally strengthening cell-wall lignification to reinforce structural defense [33].

#### 2.5.1. EPSs Increase SOD Activity During TMV Infection

**Figure 4:**
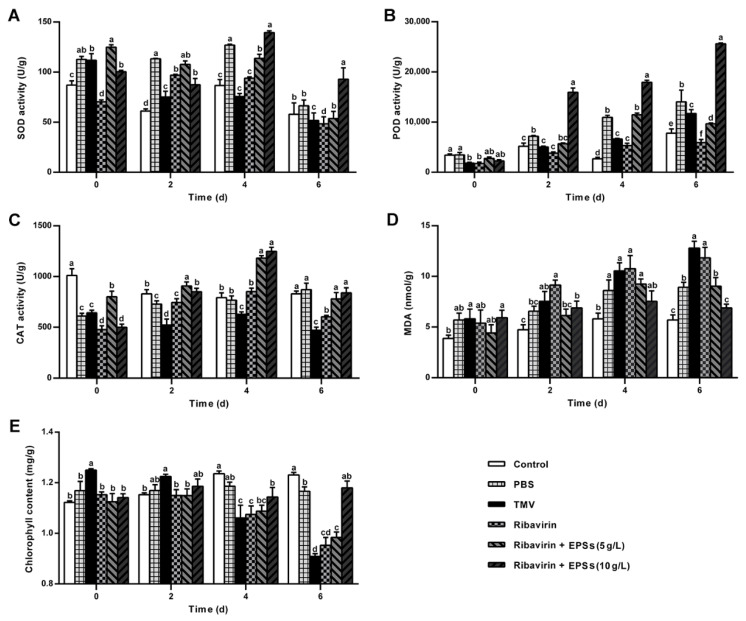
Effects of EPSs on antioxidant enzyme activities, lipid peroxidation, and chlorophyll content in *N. benthamiana.* (**A**) SOD activity. (**B**) POD activity. (**C**) CAT activity. (**D**) MDA concentration. (**E**) Chlorophyll content. To assess treatment effects at each time point, two-way ANOVA was performed, and the corresponding MSE and degrees of freedom were used to recalculate critical differences prior to assigning significance. Tukey’s HSD test, accounting for time × treatment interactions, was applied for multiple comparisons; detailed statistical outputs are provided in Figure S3. Different letters (a, b, c, d) in the figure indicate significance levels. The same letter denotes no significant difference (*p* > 0.05), while different letters indicate a significant difference (*p* < 0.05).

As shown in Figure 4A, EPSs markedly modulated SOD activity following TMV inoculation. While the TMV group showed a continuous decline from Day 0–6, the 10 g/L EPS treatment produced the highest SOD activity on Day 4 (1.39-fold over baseline), surpassing both Ribavirin alone and the combined 5 g/L EPSs treatment. This enzyme upregulation resembles previously reported induction of host defense enzymes by citral [34]. Although SOD activity decreased by Day 6 across all treatments, the 10 g/L EPS group remained significantly higher than all other groups (*p* < 0.05), indicating that EPSs enhance early ROS-scavenging capacity during TMV infection.

#### 2.5.2. EPSs Enhance POD Activity and Reinforce Defense

POD activity exhibited a distinct response pattern (Figure 4B). While Control and PBS groups showed increases likely attributable to mechanical injury or minor environmental fluctuations, TMV inoculation resulted in a rise-and-fall trajectory. Ribavirin, EPSs (5 g/L), and EPSs (10 g/L) all elevated POD activity, reaching maxima at Day 6, 4, and 6, respectively. Peak POD levels in the 5 and 10 g/L EPS groups were 4.16-fold and 11.37-fold higher than baseline—significantly exceeding both Control and TMV groups (*p* < 0.05). Importantly, POD activity in the 10 g/L EPS group continued rising through Day 6 and remained significantly higher than other treatments (*p* < 0.05), demonstrating that EPSs strongly promote POD-associated defense. This pattern parallels previous observations in Luo et al. [34], where limonene induced substantial POD activation, supporting the notion that plant-derived polysaccharides or related metabolites can enhance enzymatic resistance responses.

#### 2.5.3. EPSs Stimulate CAT Activity to Mitigate Oxidative Stress

CAT activity also responded positively to EPS treatment (Figure 4C). While control plants showed minimal fluctuation and PBS treatment induced a mild rise, TMV infection caused divergent responses across treatment groups. CAT activity in Ribavirin, 5 g/L EPSs, and 10 g/L EPS groups increased markedly, with all peaking at Day 4. The 10 g/L EPS treatment again produced the most pronounced enhancement, reaching 2.5-fold above baseline and significantly exceeding both Ribavirin and 5 g/L EPSs (*p* < 0.05). Although CAT activity declined by Day 6 in all TMV-inoculated groups, the 10 g/L EPS group remained comparable to the control (*p* > 0.05). This strong CAT induction contrasts with prior findings in which salicylic acid (SA) suppressed CAT activity [35], suggesting that EPSs may modulate CAT through alternative signaling pathways that warrant further investigation.

#### 2.5.4. EPSs Reduce Lipid Peroxidation (MDA)

In addition to antioxidant enzymes, EPSs also affected lipid peroxidation levels (Figure 4D). TMV infection caused a sharp rise in MDA content in the TMV group, reaching 12.80 nmol/g at Day 6. Ribavirin alone failed to mitigate this increase. EPSs substantially reduced MDA accumulation, particularly at 10 g/L, where MDA increased only 1.16-fold compared with baseline (6.88 nmol/g), significantly lower than Ribavirin and 5 g/L EPS groups (*p* < 0.05). These results indicate that EPSs effectively suppresses TMV-induced membrane lipid peroxidation [36].

#### 2.5.5. EPSs Maintain Chlorophyll Content Under Viral Stress

Finally, EPSs also preserved chlorophyll levels during TMV infection (Figure 4E). The Control group maintained stable chlorophyll content, while TMV caused a rapid decline from Day 2–6. Ribavirin alone or with 5 g/L EPSs did not prevent chlorophyll loss. In contrast, 10 g/L EPSs maintained chlorophyll at pre-inoculation levels, significantly differing from TMV, Ribavirin, and 5 g/L EPS groups (*p* < 0.05). TMV-induced chlorophyll reduction is consistent with reported viral disruption of chloroplast-associated defense pathways [37]. Thus, EPSs appear to counteract TMV-mediated chloroplast damage, likely via enhanced antioxidant protection and strengthened host defense, ultimately improving plant resistance.

### 2.6. EPSs Modulate Defense-Related Gene Expression in N. benthamiana

Plant exposure to pathogens triggers multiple defense responses, including ROS accumulation [38], phytohormone signaling [39], production of antimicrobial metabolites [40], and activation of pathogenesis-related (PR) proteins [41]. PR proteins serve as biochemical markers of SA signaling; their rapid accumulation reflects pathogen- or SA-induced activation of the pathway [41,42]. Plants regulate these responses by adjusting signaling molecule levels, modulating defense-related gene expression, and coordinating complex crosstalk between pathways. In this study, we investigated the expression of key genes involved in these pathways, including nonexpressor of pathogenesis-related genes 1 (*NPR1*), pathogenesis-related protein 1 (*PR1*), Coronatine Insensitive 1 (*COI1*), Ethylene Response Factor 1 (*ERF1*), Respiratory Burst Oxidase Homolog B (*RBOHB*), Phenylalanine ammonia-lyase (*PAL*), and Pyruvate Oxidase-Related 1 (*POR1*), to evaluate the effects of EPS treatment.

#### 2.6.1. EPSs Induce SA Pathway Gene Expression

SA is a key phytohormone regulating plant innate immunity and broad-spectrum resistance against viruses, bacteria, and fungi [43]. SA mediates SAR via NPR receptor proteins and activates downstream genes such as *PR1* [44].

**Figure 5:**
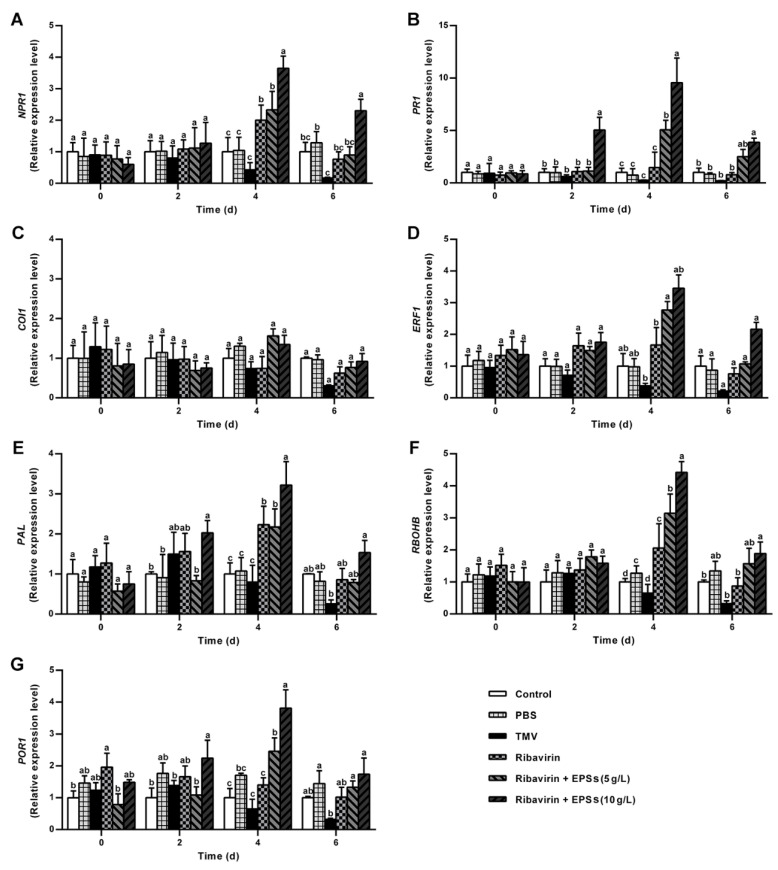
Effects of EPSs on the expression of defense-related genes in *N. benthamiana*. (**A**) *NPR1*. (**B**) *PR1*. (**C**) *COI1*. (**D**) *ERF1*. (**E**) *RBOHB*. (**F**) *PAL*. (**G**) *POR1*. Gene expression levels were quantified by qRT-PCR. Data represent means ± SD of three biological replicates. Two-way ANOVA was used to evaluate treatment effects at each time point, and the corresponding mean square error and degrees of freedom were applied to recalculate critical differences before assigning significance. Tukey’s HSD test, accounting for time × treatment interactions, was used for post hoc comparisons; detailed statistical outputs are provided in Figure S4. Different letters (a, b, c, d) in the figure indicate significance levels. The same letter denotes no significant difference (*p* > 0.05), while different letters indicate a significant difference (*p* < 0.05).

As shown in Figure 5A, EPS treatment significantly upregulated *NPR1* expression in *N. benthamiana.* On Day 4 post treatment, Ribavirin, 5 g/L EPS and 10 g/L EPS groups exhibited significant upregulation compared with the Control (*p* < 0.05), with the 10 g/L EPS group reaching 6.07-fold of the baseline. On Day 6, *NPR1* levels declined slightly in all groups but remained significantly elevated in the 10 g/L EPS group (3.83-fold), whereas TMV group expression decreased to 0.19-fold.

Similarly, *PR1* expression (Figure 5B) was significantly induced by EPSs, with the 10 g/L EPS group reaching 11.00-fold on Day 4, indicating that EPSs activate SA-dependent defense, likely through *NPR1*-mediated SAR induction. This effect is consistent with previous reports of exogenous SA treatment enhancing *NPR1* and *PR1* expression to confer disease resistance [44,45].

#### 2.6.2. EPSs Modulate Jasmonic Acid (JA) Pathway Gene Expression

JA regulates plant stress responses, including stomatal closure, root growth, chloroplast development, and photosynthesis [46]. *COI1* functions as the central receptor in the JA pathway, perceiving JA and triggering downstream defenses [47]. As shown in Figure 5C, *COI1* expression in EPS-treated groups (5 g/L and 10 g/L) was upregulated on Day 4 (1.93- and 1.58-fold of baseline, respectively), while TMV and Ribavirin groups showed a decline. By Day 6, *COI1* expression decreased in all groups, with the lowest level in the TMV group (0.31-fold). The muted effect of EPSs on *COI1* may reflect antagonism between SA and JA signaling [48], suggesting that EPSs preferentially activate SA-dependent defenses.

#### 2.6.3. EPSs Enhance Ethylene (ET) Pathway Gene Expression

ET signaling, mediated by *ERF1*, coordinates plant defense and development [49]. As shown in Figure 5D, 10 g/L EPS treatment increased *ERF1* expression, reaching 2.54-fold on Day 4 and maintaining 1.59-fold on Day 6. This induction likely contributes to enhanced PR gene expression and ROS-mediated defense, consistent with reports of ERF factors regulating stress responses via PR proteins and ROS signaling [50,51,52].

#### 2.6.4. EPSs Upregulate Phenylpropanoid Pathway Genes

PAL is a key enzyme in the phenylpropanoid pathway, contributing to SA biosynthesis and secondary metabolites associated with plant defense [53,54]. As shown in Figure 5E, *PAL* expression in the 10 g/L EPS-treated group increased progressively, peaking on Day 4 (4.29-fold of baseline), whereas the TMV group expression declined. EPS-induced *PAL* upregulation likely promotes SA accumulation, further activating the SA pathway and enhancing resistance [55,56].

#### 2.6.5. EPSs Trigger ROS Burst Pathway Genes

ROS accumulation is a key defense response that activates SAR but can be damaging if excessive [57]. *RBOHB* is an ROS-generating enzyme crucial for initiating oxidative bursts [58]. As shown in Figure 5F, EPS treatment (5 g/L and 10 g/L groups) induced significant *RBOHB* upregulation on Day 4, reaching 4.43-fold in the 10 g/L EPS group. Enhanced SOD, POD, and CAT activities likely mitigate ROS toxicity while maintaining defense signaling, demonstrating that EPSs promote an early ROS burst to strengthen plant immunity [59].

#### 2.6.6. EPSs Stabilize Chlorophyll Biosynthesis Gene Expression

*POR1* is a key enzyme in chlorophyll biosynthesis [60,61]. TMV infection suppresses *POR1*, leading to chlorophyll degradation. As shown in Figure 5G, 10 g/L EPS treatment maintained *POR1* expression up to 2.57-fold on Day 4, counteracting TMV-induced suppression in TMV and Ribavirin groups. This suggests that EPSs help preserve chlorophyll biosynthesis, maintaining photosynthetic capacity and enhancing plant resilience under viral attack.

Taken together, the biological activities observed for the EPSs should be interpreted with careful consideration of the applied purification strategy, compositional characteristics, and the current level of structural resolution. The EPSs were obtained through ethanol precipitation, deproteinization, and subsequent Sephadex LH-20 gel filtration, yielding glucose-rich, gel-filtration–fractionated polysaccharide preparations, rather than fully homogeneous single macromolecules. Compositional analysis indicated a high glucose content (97.67%), accompanied by minor amounts of protein, uronic acid, and sulfate (1.18%). Notably, despite this relatively low degree of sulfation, sulfate groups may play a crucial role in mediating the antiviral activity of the EPSs. Accumulating evidence has demonstrated a significant positive correlation between the degree of sulfation (DS) of polysaccharides and their antiviral potency: specifically, polysaccharides such as fucoidan, carrageenan, sulfated glucan, pectin derivatives, and arabinoxylan exhibit lower half-maximal inhibitory concentrations (IC_50_) with increasing DS values [62,63,64]. Nevertheless, it is undeniable that the glucan backbone can also confer a baseline antiviral activity, which may be associated with its spatial conformation, molecular weight, and the presence of functional groups (e.g., carboxyl or aldehyde groups) [63,65]. With respect to antioxidant activity, previous studies have shown that sulfation can significantly enhance free radical-scavenging capacity and reduce the power of polysaccharides, while non-sulfated polysaccharides (e.g., native glucans and pectin) often possess considerable inherent antioxidant activity on their own [62,66].

Comprehensive structural elucidation, including FTIR spectroscopy, nuclear magnetic resonance (^1^H, ^13^C NMR) [67], or methylation-based glycosidic linkage analysis [68], was not performed in the present study. Accordingly, the EPSs should be regarded as functionally defined, glucose-rich polysaccharide fractions rather than fully structurally resolved individual molecules. In this study, all antiviral assays were conducted using the pooled total EPS fraction obtained after gel filtration, and the specific contributions of individual molecular-weight fractions to the observed activities were not independently assessed. Therefore, the antiviral and antioxidant effects reported herein reflect the integrated biological activity of the EPS mixture as a whole, while the relative activity of distinct molecular-weight components remains to be elucidated in future investigations. Future studies will incorporate detailed glycosidic linkage analysis to further clarify structure–activity relationships.

Considering the relatively high average molecular weight of the EPSs (3.56 × 10^4^ Da), the mass concentrations applied in the antiviral assays (5 g/L and 10 g/L) correspond to molar concentrations of approximately 0.14 mM and 0.28 mM, respectively. These values are within a comparable order of magnitude to that of the small-molecule antiviral control ribavirin (0.5 g/L, 2.05 mM) [69,70]. The literature reports on other natural polysaccharide-based biostimulants—including Lentinan and sulfated Lentinan (high-molecular-weight polysaccharides, effective at 2.5-10 μg/mL) [11], burdock-derived fructooligosaccharides (2.13 kDa, 5 g/L) [12], and DNPE6(4) (99.2 kDa, 125 μg/mL) [13]—which demonstrates that effective mass concentrations generally scale with molecular size and structural complexity. Within this context, the EPS dosage employed in the present study is reasonable for a natural macromolecular polysaccharide system.

While detailed structural comparisons with other antiviral fungal glucans (e.g., lentinan) were not performed, the monosaccharide composition and molecular size of the EPSs suggest potential functional similarities. Collectively, these considerations integrate purification strategy, compositional features, molecular weight, and dosage rationale, providing a comprehensive explanation for the bioactivities of the EPSs in *N. benthamiana*. This work thus lays a foundation for further exploration of the EPSs’ application potential, while delineating clear priorities for future structural characterization and mechanism-focused studies.

## 3. Materials and Methods

### 3.1. Materials

#### 3.1.1. Strains, Virus, and Plants

The fungal strain *Beauveria bassiana* T2-2 was isolated from volcanic sediments in the East Pacific and deposited at the Marine Culture Collection of China (MCCC, accession No. 3A01545).

Tobacco mosaic virus (TMV) inoculum was extracted from infected Yunyan 87 tobacco leaves provided by Fujian China Tobacco Industry Co., Ltd. (Xiamen, China) and stored at −80 °C until use.

*Nicotiana benthamiana* was used as the host plant, a widely adopted model for plant virology studies due to its susceptibility to various plant viruses.

#### 3.1.2. Culture Media

Potato Dextrose Agar (PDA) was prepared by dissolving 24 g of potato dextrose broth (Huankai Microbial, Guangzhou, China) in 1 L ultrapure water, adding 15 g agar, mixing gently, sterilizing at 121 °C for 20 min, and pouring into plates. Potato Dextrose Broth (PDB) was prepared similarly to PDA, but without the addition of agar.

The fermentation medium was prepared by dissolving 20 g glucose, 2 g peptone, 1.5 g KH_2_PO_4_, 1.0 g MgCl_2_, 0.1 g KCl, and 0.5 g CaCl_2_ in 1 L ultrapure water, followed by pH adjusting to 8.0. The medium was sterilized at 115 °C for 20 min, rather than the conventional 121 °C, to minimize glucose caramelization and Maillard reactions between glucose and amino acids from peptone, which can occur at higher temperatures and adversely affect nutrient availability [71]. This temperature–time combination ensured effective sterilization while preserving medium quality [72,73], and co-autoclaving glucose with peptone at 115 °C did not result in observable caramelization or impaired fungal growth in preliminary experiments.

### 3.2. EPS Extraction and Characterization

#### 3.2.1. EPS Production and Purification

A single colony of *B. bassiana* T2-2 was inoculated into sterilized PDB and cultured at 28 °C with shaking at 180 rpm for 2 days to obtain seed culture. Subsequently, 2% (*v*/*v*) of the seed culture was transferred to fresh PDB and fermented under the same conditions for 7 days. Although spore counting is commonly used to standardize fungal inoculum, this volume-based inoculation strategy was adopted because it yielded reproducible EPS production under the defined laboratory-scale conditions used in this study and is commonly applied in preliminary fungal fermentation experiments.

After fermentation, the culture broth was centrifuged at 10,000 rpm, 4 °C for 15 min to remove mycelia and insoluble impurities. The supernatant was concentrated under reduced pressure at 40 °C to 1/10 of the original volume, followed by precipitation with four volumes of pre-cooled absolute ethanol and incubation at 4 °C overnight. The precipitate was collected by centrifugation, pre-frozen for 12 h, and freeze-dried to obtain crude EPS powder.

To further remove low-molecular-weight impurities, including residual monosaccharides and small metabolites, the crude EPS was dissolved in distilled water and subjected to Sephadex LH-20 gel (Cytiva, Uppsala, Sweden) filtration chromatography. Sephadex LH-20 was selected due to its effectiveness in separating polysaccharides from low-molecular-weight carbohydrates based on size exclusion principles [22].

Elution was performed with distilled water, and fractions were collected sequentially. The collected fractions were monitored by thin-layer chromatography (TLC), and only fractions corresponding to polysaccharide components were pooled, concentrated, and lyophilized. The resulting gel-filtration–fractionated EPSs were used for molecular weight determination, monosaccharide composition analysis, and all subsequent biological activity assays.

#### 3.2.2. Physicochemical Analysis of EPSs

Total Sugar Content: The total sugar content of EPSs was determined using the phenol–sulfuric acid method, with glucose as the standard. This spectrometric assay was employed to provide a rapid and reliable quantification of total carbohydrate content in the EPS samples. Briefly, an EPS solution (0.1 mg/mL) was reacted with phenol and concentrated sulfuric acid, heated in a boiling water bath for 10 min, and cooled, undergoing absorbance measured at 490 nm. The use of this method complements the HPLC-based monosaccharide composition analysis, which was performed separately after hydrolysis to resolve individual monosaccharide components.

Protein Content: It was assessed using the Coomassie Brilliant Blue method with bovine serum albumin (BSA) as standard. EPS solution (10 mg/mL) was mixed with dye reagent and absorbance measured at 595 nm.

Uronic Acid Content: It was determined by the sulfuric acid–carbazole method using galacturonic acid (GalA) as standard, with absorbance measured at 530 nm.

Sulfate Content: It is measured by the barium chloride–gelatin turbidimetric method after acid hydrolysis at 100 °C for 8 h; absorbance determined at 360 nm.

Molecular Weight: It was determined by gel permeation chromatography (GPC) using TSK Gel GMPWxl column with ultrapure water as mobile phase, 0.5 mL/min flow rate, and differential refractive index detector. Dextran standards (2 × 10^3^, 5 × 10^3^, 1 × 10^4^, 2 × 10^4^ and 5 × 10^4^ Da) were used for calibration.

Monosaccharide Composition: It was analyzed by 1-phenyl-3-methyl-5-pyrazolone (PMP) pre-column derivatization HPLC. EPSs were hydrolyzed with trifluoroacetic acid (TFA), derivatized with PMP, extracted with chloroform, filtered, and injected onto a Waters Xbridge Prep C18 column, detected at 250 nm (Table S1).

### 3.3. Optimization of EPS Fermentation Conditions

#### 3.3.1. Fermentation Parameters

Effects of rotation speed (140, 160, 180, 200 and 220 rpm), temperature (24, 26, 28, 30 and 32 °C), initial pH (5, 6, 7, 8 and 9), inoculum size (2, 4, 6, 8 and 10%), and fermentation duration (3, 5, 7, 9 and 11 days) on EPS yield were evaluated using fermentation medium. The yield of EPSs produced by *B. bassiana* T2-2 was used as the primary criterion for optimizing fermentation conditions.

#### 3.3.2. Medium Composition

Carbon and nitrogen sources as well as inorganic salts were screened in single-factor experiments: the carbon sources (20 g/L; Macklin, Shanghai, China) comprised glucose, lactose, sucrose, soluble starch, dextrin, and mannose; nitrogen sources (2 g/L; Macklin, Shanghai, China) included peptone, yeast extract, NH_4_NO_3_, (NH_4_)_2_SO_4_, NH_4_Cl, and beef extract; and the inorganic salts (1 g/L; Macklin, Shanghai, China) evaluated were KH_2_PO_4_, MgCl_2_, ZnSO_4_, CuSO_4_, Na_2_SO_4_, and CaCl_2_.

#### 3.3.3. Orthogonal Optimization

Based on single-factor experiments, lactose (carbon source), peptone (organic nitrogen source), (NH_4_)_2_SO_4_ (inorganic nitrogen source), and Na_2_SO_4_ (inorganic salt) were selected for L9 (3^4^) orthogonal experiments to determine optimal proportions.

### 3.4. TMV Inoculum Preparation

#### 3.4.1. Crude Virus Extraction

Frozen TMV-infected tobacco leaves were ground in liquid nitrogen, homogenized in PBS (0.01 M, pH 7.2, 1% 2-mercaptoethanol), filtered, and subjected to n-butanol precipitation and PEG-6000/NaCl precipitation. Virus pellets were resuspended in PBS, flash-frozen with liquid nitrogen, and stored at −80 °C.

#### 3.4.2. Virus Quantification and Validation

Virus concentration was determined by absorbance at 260 nm. RNA extraction was performed using Trizol, followed by reverse transcription using HiScript III All-in-one RT SuperMix (Vazyme, Nanjing, China). TMV cDNA was amplified using specific primers (GenBank AF273221.1, Table S2) and confirmed by sequencing. Virus morphology was verified by TEM after negative staining with 2% phosphotungstic acid.

### 3.5. TMV Infection Model in N. benthamiana

#### 3.5.1. Plant Cultivation

Seeds were sown in peat soil, covered for moisture retention, and seedlings were transplanted at the three-leaf stage. Plants were maintained at 23–27 °C, 60–70% humidity, under a 16/8 h light/dark cycle.

#### 3.5.2. Virus Inoculation

Mechanical inoculation was performed on 4–5-week-old plants using carborundum as an abrasive. Virus concentration was adjusted to 100 μg/mL in PBS. Inoculated leaves were rinsed after 30 min. Disease progression was recorded daily according to standardized scoring.

#### 3.5.3. EPS Treatment

Six treatment groups were established according to the applied regimen: Control (healthy plants without any treatment), PBS (mock inoculation with phosphate-buffered saline instead of TMV), TMV (challenge inoculation with 100 µg/mL TMV), Ribavirin (TMV inoculation followed by 500 µg/mL ribavirin spray), Ribavirin + EPSs (5 g/L) (TMV inoculation followed by combined application of ribavirin and 5 g/L EPSs), and Ribavirin + EPSs (10 g/L) (TMV inoculation followed by combined application of ribavirin and 10 g/L EPSs).

### 3.6. Defense Enzyme and Chlorophyll Measurements

Leaf samples were collected at 0, 2, 4, and 6 days, frozen in liquid nitrogen, and stored at −80 °C. Crude enzyme extracts were prepared and activities of SOD, POD, CAT, MDA content, and chlorophyll content were measured using commercial assay kits (Solarbio, Beijing, China).

### 3.7. RNA Extraction and RT-qPCR

Leaf tissues were ground under liquid nitrogen, and total RNA was extracted following Trizol-based protocols. cDNA was synthesized from 2 μg RNA using HiScript III kit (Vazyme, China). RT-qPCR was performed using TB Green^®^ Premix Ex Taq™ (Taraka, Beijing, China) with specific primers (Table S3), including internal reference genes, under standard cycling conditions (pre-denaturation 95 °C 30 s; 40 cycles of 95 °C 10 s, 50 °C 15 s; melt curve 60–95 °C). Three biological and technical replicates were performed.

### 3.8. Data Analysis

Except for the inorganic salt matrix, all optimization experiments were analyzed by Dunnett’s test against the original fermentation condition as the control. Inorganic salt data were subjected to one-way ANOVA followed by Tukey’s HSD post hoc test for pairwise comparisons.

Enzyme-activity and gene-expression datasets were evaluated by two-way ANOVA with Tukey’s HSD correction. For single time point comparisons among treatments, a two-way ANOVA was first performed; the mean square error (MSE) and degrees of freedom (df) were then used to recalculate the critical difference (CD) before significance assignment.

All statistical analyses and graphics were generated with GraphPad Prism 8.0. Significant differences are indicated by lowercase letters: treatments sharing at least one letter are not significantly different (*p* ≥ 0.05), whereas those with no letter in common differ significantly.

## 4. Conclusions

The extracellular polysaccharides (EPSs) from deep-sea *Beauveria bassiana* T2-2 was efficiently produced under optimized fermentation conditions, yielding 3.42 g/L. EPSs exhibited strong protective effects against TMV in *Nicotiana benthamiana*, alleviating typical viral symptoms. Mechanistically, EPSs enhanced antioxidant enzyme activities (SOD, POD, CAT), reduced lipid peroxidation, maintained chlorophyll content, and upregulated defense-related genes in salicylic acid, ethylene, and phenylpropanoid pathways, promoting ROS-mediated antiviral responses. Combined treatment with Ribavirin further improved these effects. This study indicates that the EPSs exhibit antiviral activity in *N. benthamiana*, supporting their potential application as polysaccharide-based biostimulants in sustainable plant protection strategies.

## Figures and Tables

**Table 1:** Orthogonal design and results of EPS fermentation optimization.

	Factor				EPSs Yield (g/L)
	Lactose (g/L)	Peptone (g/L)	(NH_4_)_2_SO_4_ (g/L)	Na_2_SO_4_ (g/L)	
1	1	1	1	1	2.108
2	1	2	2	2	2.528
3	1	3	3	3	2.757
4	2	1	2	3	2.563
5	2	2	3	1	3.253
6	2	3	1	2	3.085
7	3	1	3	2	2.669
8	3	2	1	3	2.919
9	3	3	2	1	3.142
k1	2.464	2.447	2.704	2.834	
k2	2.967	2.900	2.744	2.761	
k3	2.910	2.995	2.893	2.746	
Range (R)	0.503	0.548	0.189	0.088	
Optimal formulation	2	3	3	1	

## Data Availability

The original contributions presented in this study are included in the article/Supplementary Material. Further inquiries can be directed to the corresponding author.

## Supplementary Materials

The following supporting information can be downloaded at https://www.mdpi.com/article/10.3390/md24010039/s1; Figure S1. HPLC chromatograms of 1-phenyl-3-methyl-5-pyrazolone (PMP)-derivatized monosaccharide standards; Figure S2. Thin-layer chromatography (TLC) analysis of fractions obtained from Sephadex LH-20 gel filtration of EPSs; Figure S3. Effects of EPSs on antioxidant enzyme activities, lipid peroxidation, and chlorophyll content in *N. benthamiana* (Tukey’s HSD); Figure S4. Effects of EPSs on the expression of defense-related genes in *N. benthamiana* (Tukey’s HSD); Table S1. Retention times of monosaccharide standards used for compositional analysis; Table S2. Oligonucleotide primers used for TMV; Table S3. Oligonucleotide primers used for qPCR analysis of defense-related marker genes in *N. benthamiana*.

## Abbreviations

The following abbreviations are used in this manuscript:

BSA, bovine serum albumin; CAT, catalase; CD, critical difference; *COI1*, Coronatine Insensitive 1; df, degrees of freedom; DS, degree of sulfation; EPSs, extracellular polysaccharides; *ERF1*, Ethylene Response Factor 1; ET, ethylene; GPC, gel permeation chromatography; HPGPC, high-performance gel permeation chromatography; HPLC, high-performance liquid chromatography; HR, hypersensitive response; IC_50_, half-maximal inhibitory concentrations; JA, jasmonic acid; MSE, mean square error; Mw, molecular weights; NJ, neighbor-joining; NP*R1*, Nonexpressor of pathogenesis-related genes 1; *PAL*, Phenylalanine ammonia-lyase; PDA, potato dextrose agar; PDB, potato dextrose broth; PMP, 1-phenyl-3-methyl-5-pyrazolone; POD, peroxidase; *POR1*, Pyruvate oxidase-related 1; PR, pathogenesis-related; *PR1*, pathogenesis-related protein 1; *RBOHB*, respiratory burst oxidase Homolog B; ROS, reactive oxygen species; SA, salicylic acid; SAR, systemic acquired resistance; SOD, superoxide dismutase; TFA, trifluoroacetic acid; TLC, thin-layer chromatography; TMV, tobacco mosaic virus.
