# Supplementary material for: Discovery of a Marine Beauveria bassiana Polysaccharide with Antiviral Activity Against Tobacco Mosaic Virus
Source: Mar Drugs. 2026 Jan 13;24(1):39. doi: 10.3390/md24010039 (PMC12842688; doi:10.3390/md24010039)
Supplement: Supplementary file 1 [file marinedrugs-24-00039-s001.zip › marinedrugs-4020116-supplementary.pdf]

Supplementary Materials

Discovery of a Marine *Beauveria bassiana* Polysaccharide with Antiviral Activity Against Tobacco Mosaic Virus

Xu Qiu [1, †], Lihang Jiao [1, 2, †], Jingjing Xue [1], Guangxin Xu [1], and Xixiang Tang [1, *]

[1] Key Laboratory of Marine Genetic Resources, Third Institute of Oceanography, Ministry of Natural Resources, Xiamen 361005, China;

[2] College of Ocean Food and Biological Engineering, Jimei University, Xiamen 361021, China.

[†] These authors contributed equally to this work.

* Correspondence: tangxixiang@tio.org.cn.

Figure S1.

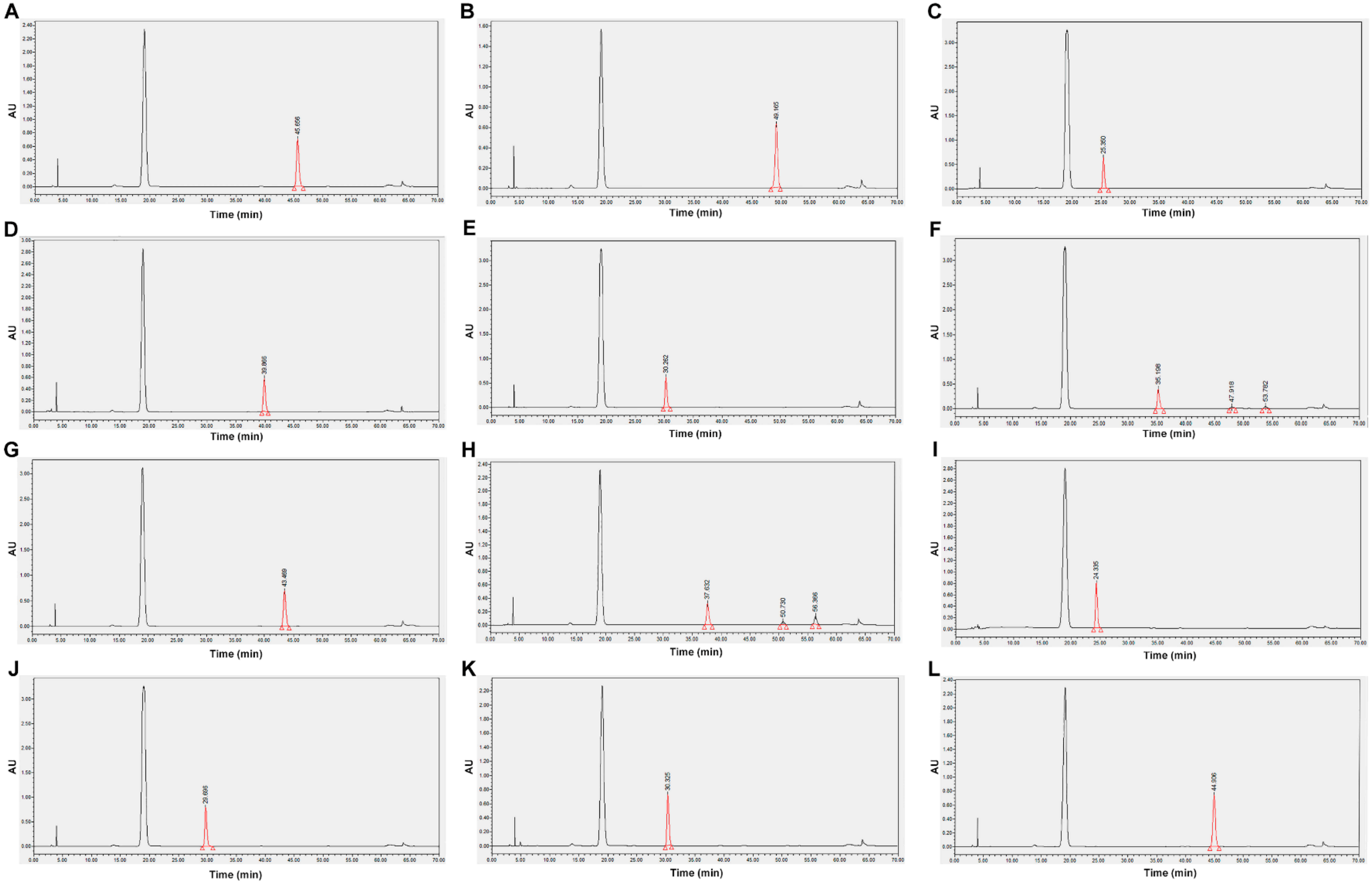

Figure S1. HPLC chromatograms of 1-phenyl-3-methyl-5-pyrazolone (PMP)-derivatized monosaccharide standards.

(A-L) Representative chromatograms of twelve monosaccharide standards: (A) Arabinose, (B) Fucose, (C) Gulose, (D) Glucose, (E) Glucosamine, (F) Glucuronic acid, (G) Galactose, (H) Galacturonic acid, (I) Mannose, (J) Ribose, (K) Rhamnose, and (L) Xylose. The retention times of each standard were used to identify the corresponding peaks in the EPSs chromatogram.

Figure S2.

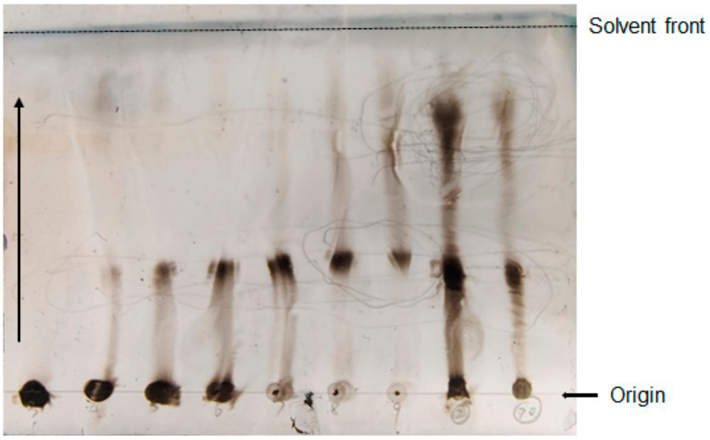

Figure S2. Thin-layer chromatography (TLC) analysis of fractions obtained from Sephadex LH-20 gel filtration of EPSs.

TLC was used to monitor fraction distribution, and fractions showing signals retained near the origin, characteristic of polysaccharide components, were pooled for subsequent analyses.

Figure S3.

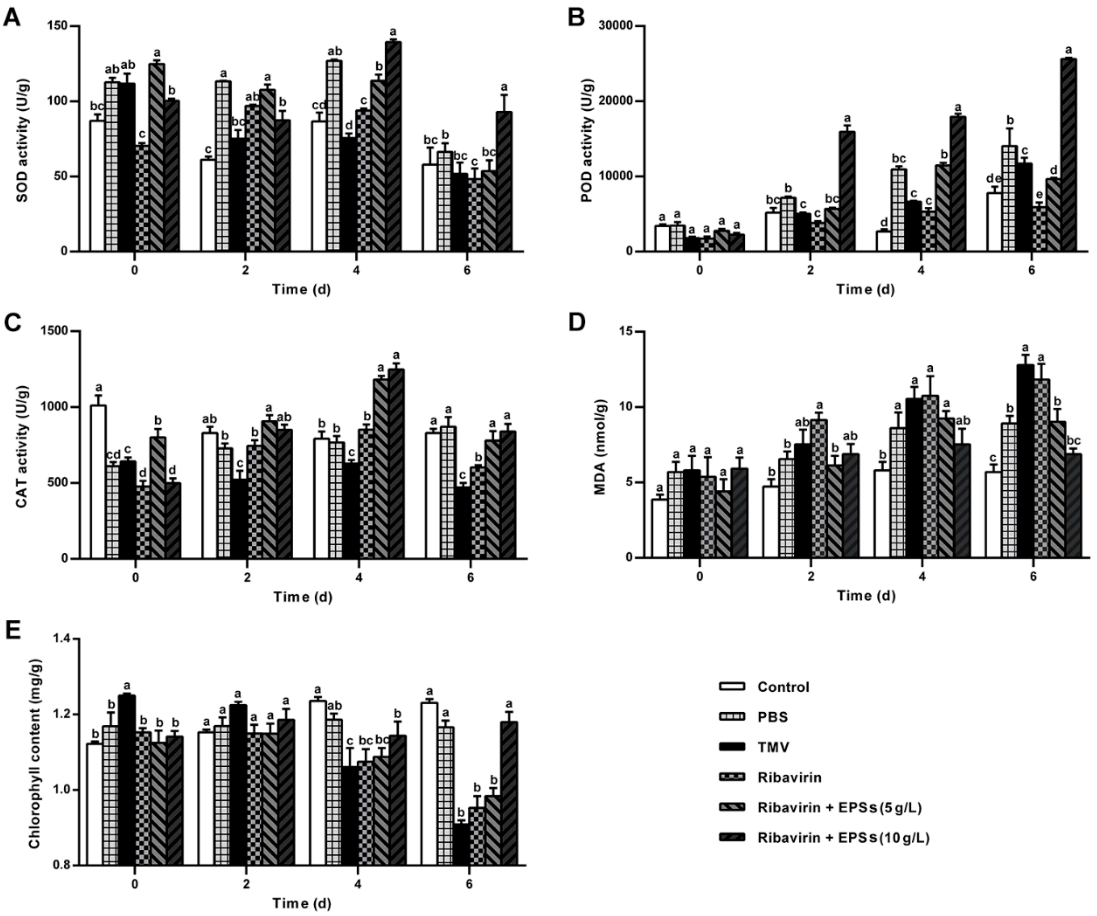

Figure S3. Effects of EPSs on Antioxidant Enzyme Activities, Lipid Peroxidation, and Chlorophyll Content in *N. benthamiana* (Tukey's HSD).

(A) SOD activity. (B) POD activity. (C) CAT activity. (D) MDA concentration. (E) Chlorophyll content. A two-way ANOVA was performed to evaluate the effects of time and treatment, followed by Tukey's HSD test that accounted for time × treatment interactions for multiple comparisons. Detailed statistical outputs are provided in this figure. Different letters (a, b, c, d) in the figure indicate significance levels. The same letter denotes no sig-nificant difference ($p > 0.05$), while different letters indicate a significant difference ($p < 0.05$).

Figure S4.

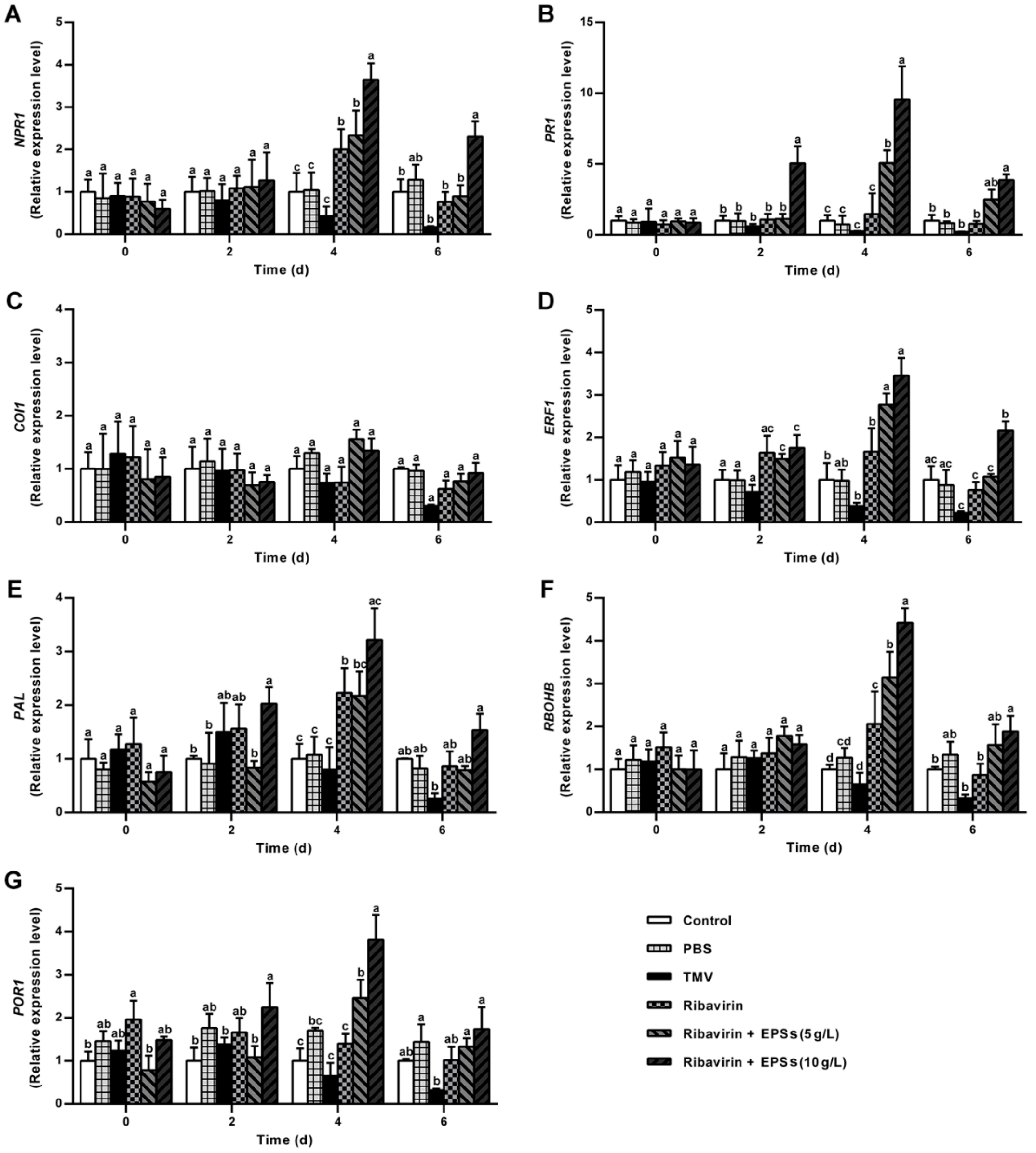

Figure S4. Effects of EPSs on the Expression of Defense-Related Genes in *N. benthamiana* (Tukey's HSD).

(A) *NPR1*. (B) *PR1*. (C) *COI1*. (D) *ERF1*. (E) *RBOHB*. (F) *PAL*. (G) *POR1*. Gene expression levels were quantified by qRT-PCR. Data represent means ± SD of three biological replicates. A two-way ANOVA was performed to evaluate the effects of time and treatment, followed by Tukey's HSD test that accounted for time × treatment interactions for multiple comparisons. Detailed statistical outputs are provided in this figure. Different letters (a, b, c, d) in the figure indicate significance levels. The same letter denotes no sig-nificant difference ($p > 0.05$), while different letters indicate a significant difference ($p < 0.05$).

Table S1. Retention times of monosaccharide standards used for compositional analysis.

Carbohydrate	Retention time (min)
Arabinose	45.656
Fucose	49.165
Gulose	25.350
Glucose	39.866
Glucosamine	30.262
Glucuronic acid	35.198
Galactose	43.469
Galacturonic acid	37.632
Mannose	24.335
Ribose	29.696
Rhamnose	30.325
Xylose	44.906

Table S2. Oligonucleotide primers used for TMV.

Gene	Sequence (5′-3′)
126 kDa subunit	F: CAATTACCAACAACAACAAACAACAAACAAC
	R: CTATTGTGTTCCTGCATCGACCTTATAC
54 kDa subunit	F: ATGCAGTTTTACTATGATAAGTGTCTCCC
	R: GAACTTTATCAGACAAATACTTCACCAGAC
MP	F: ATGGCTCTAGTTGTTAAAGGAAAAGTGAATATC
	R: GAATCCGATTCGGCGACAGTAG
CP	F: TAGACCCGCTAGTCACAG
	R: CAGAGGTCCAAACCAAAC

Table S3. Oligonucleotide primers used for qPCR analysis of defense-related marker genes in *N. benthamiana.*

Gene	Sequence (5′-3′)
β-actin	F: AGGGTTTGCTGG AGATGATG
	R: CGGGTTAAGAGGTGCTTCAG
NPR1	F: ACATCAGCGGAAGCAGTAG
	R: GTCGGCGAAGTAGTCAAAC
PR1	F: CCTCGTACATTCTCATGGTCAAT
	R: CCATTGTTACACTGAACCCTAGC
COI1	F: CAGCAGCCCATTGTTTCTTAC
	R: TACTGGCCAAGTACTTCCAATC
ERF1	F: GCTCTTAACGTCGGATGGTC
	R: AGCCAAACCCTAGCTCCATT
PAL	F: GTTATGCTCTTAGAACGTCGCCC
	R: CCGTGTAATGCCTTGTTTCTTGA
RBOHB	F: TTTTCTCTGAGGTTTGCCAGCCACCA
	R: GCCTTCATGTTGTTGACAATGTCTTT
POR1	F: GGACCCACAAGCTATTCCCA
	R: GGACCCACAAGCTATTCCCA
